# Conservation tillage: a way to improve yield and soil properties and decrease global warming potential in spring wheat agroecosystems

**DOI:** 10.3389/fmicb.2024.1356426

**Published:** 2024-06-04

**Authors:** Mahran Sadiq, Nasir Rahim, Majid Mahmood Tahir, Abdulrahman Alasmari, Mesfer M. Alqahtani, Abdulaziz Albogami, Kholoud Z. Ghanem, Mohamed A. Abdein, Mohammed Ali, Nasir Mehmood, Jianyu Yuan, Aqila Shaheen, Muhammad Shehzad, Mohamed H. El-Sayed, Guoxiang Chen, Guang Li

**Affiliations:** ^1^College of Forestry, Gansu Agricultural University, Lanzhou, China; ^2^Department of Soil and Environmental Sciences, University of Poonch Rawalakot, Rawalakot, Pakistan; ^3^College of Grassland Science, Gansu Agricultural University, Lanzhou, China; ^4^Biology Department, College of Science, Tabuk University, Tabuk, Saudi Arabia; ^5^Department of Biological Sciences, Faculty of Science and Humanities, Shaqra University, Ad-Dawadimi, Saudi Arabia; ^6^Biology Department, Faculty of Science, Al-Baha University, Alaqiq, Saudi Arabia; ^7^Department of Biological Science, College of Science & Humanities, Shaqra University, Riyadh, Saudi Arabia; ^8^Seeds Development Department, El-Nada Misr Scientific Research and Development Projects, Turrell, Mansoura, Egypt; ^9^Maryout Research Station, Genetic Resources Department, Desert Research Center, Cairo, Egypt; ^10^College of Horticulture and the Fujian provincial Key Laboratory of Plant Functional Biology, Fujian Agricultural and Forestry University, Fuzhou, China; ^11^Department of Agronomy, University of Poonch Rawalakot, Rawalakot, Pakistan; ^12^Department of Biology, College of Sciences and Arts-Rafha, Northern Border University, Arar, Saudi Arabia

**Keywords:** climate-smart agriculture, carbon sequestration, greenhouse gases, global warming, nutrients, sustainable conservation tillage, yield

## Abstract

Climate change is one of the main challenges, and it poses a tough challenge to the agriculture industry globally. Additionally, greenhouse gas (GHG) emissions are the main contributor to climate change; however, croplands are a prominent source of GHG emissions. Yet this complex challenge can be mitigated through climate-smart agricultural practices. Conservation tillage is commonly known to preserve soil and mitigate environmental change by reducing GHG emissions. Nonetheless, there is still a paucity of information on the influences of conservation tillage on wheat yield, soil properties, and GHG flux, particularly in the semi-arid Dingxi belt. Hence, in order to fill this gap, different tillage systems, namely conventional tillage (CT) control, straw incorporation with conventional tillage (CTS), no-tillage (NT), and stubble return with no-tillage (NTS), were laid at Dingxi, Gansu province of China, under a randomized complete block design with three replications to examine their impacts on yield, soil properties, and GHG fluxes. Results depicted that different conservative tillage systems (CTS, NTS, and NT) significantly (*p* < 0.05) increased the plant height, number of spikes per plant, seed number per meter square, root yield, aboveground biomass yield, thousand-grain weight, grain yield, and dry matter yield compared with CT. Moreover, these conservation tillage systems notably improved the soil properties (soil gravimetric water content, water-filled pore space, water storage, porosity, aggregates, saturated hydraulic conductivity, organic carbon, light fraction organic carbon, carbon storage, microbial biomass carbon, total nitrogen, available nitrogen storage, microbial biomass nitrogen, total phosphorous, available phosphorous, total potassium, available potassium, microbial counts, urease, alkaline phosphatase, invertase, cellulase, and catalase) while decreasing the soil temperature and bulk density over CT. However, CTS, NTS, and NT had non-significant effects on ECe, pH, and stoichiometric properties (C:N ratio, C:P ratio, and N:P ratio). Additionally, conservation-based tillage regimes NTS, NT, and CTS significantly (*p* < 0.05) reduced the emission and net global warming potential of greenhouse gases (carbon dioxide, methane, and nitrous oxide) by 23.44, 19.57, and 16.54%, respectively, and decreased the greenhouse gas intensity by 23.20, 29.96, and 18.72%, respectively, over CT. We conclude that NTS is the best approach to increasing yield, soil and water conservation, resilience, and mitigation of agroecosystem capacity.

## 1 Introduction

Climate change is one of the main challenges, and it poses a tough challenge to the agriculture industry globally (Feng et al., 2023). Additionally, greenhouse gas (GHG) emissions are the main contributor to environmental change, and croplands are potential sources of major GHG emissions, for instance, carbon dioxide (CO_2_), methane (CH_4_), and nitrous oxide (N_2_O), that lead to increased global warming (Pu et al., 2022; Wu et al., 2022; Li et al., 2023). The agricultural sector, particularly farmlands, mainly contributes almost 12% of GHG emissions in the atmosphere (Raihan and Tuspekova, 2022). The average worldwide temperature will rise by 3.8°C by the end of the 21st century if the GHG concentrations continue to rise to the existing level (IPCC, 2021). Consequently, for achieving carbon reduction or carbon neutrality goals, the decline of emissions of major GHG is very significant to alleviate greenhouse effects in the agroecosystem. In addition, croplands have very low soil organic matter (SOM) due to environmental limitations that limit primary crop productivity (Yuan et al., 2023a). One approach to increasing soil and water conservation and crop yields while reducing greenhouse gas emissions in crop cultivation systems is to adopt climate-smart farming practices known as conservation agriculture (Ma et al., 2021; Roy et al., 2022; Li et al., 2023).

Wheat is lately one of the vigorous cereal crops with universal importance, and it has a massive influence on global food security (Huang et al., 2003). Approximately 24 million hectares’ area in China is under cultivation (Li et al., 2019). Climate change or environmental variability seriously affects crop production in the agriculture sector in China, especially in the Northwestern Loess Plateau (Alhassan et al., 2021). Furthermore, traditional or conventional tillage is the dominant crop cultivation practice in wheat cropping, generally plowing two times a year with the removal of crop straw in China, particularly in the Dingxi belt. Nevertheless, this CT practice can cause variations in soil properties (physical, chemical, biochemical, and biological) (Jat et al., 2020; Sadiq et al., 2021a; Yuan et al., 2023a), crop yield (Alhassan et al., 2021; Sadiq et al., 2021b), and greenhouse gas fluxes (Alhassan et al., 2021; Yuan et al., 2022; Feng et al., 2023) and enhance the risk of soil degradation by erosion (Liu et al., 2015; Gao et al., 2019) and global warming by greenhouse gas emissions (Alhassan et al., 2021; Feng et al., 2023). To decrease land, crop, and environmental degradation under traditional tillage measures, substantial consideration has been paid lately to soil conservation tillage as a maintainable approach for cropland ecosystems (Wulanningtyas et al., 2021; Kristine et al., 2022; Yuan et al., 2022). Soil conservation tillage is an innovative mode of modern farming that might effectively mitigate and solve the negative influences and problems of intensive tillage (Lv et al., 2023).

Conservation tillage system broadly refers to techniques employing no or less soil inversion and a minimum number of tillage operations lacking of any soil inversion and leaving at least 30% stubbles on the surface of the soil, which enhances soil and water conservation. It could be roughly divided into straw incorporation into the field, no-tillage, mulch tillage, or straw-retention with no-tillage system, minimum tillage, ridge tillage, and strip tillage (Yuan et al., 2023a). It is a well-established approach to improving soil physicochemical, microbial, and crop yields, reducing greenhouse gas emissions, mitigating negative influences of conventional tillage and climate change through soil carbon sequestering in agricultural systems, and improving agricultural and environmental sustainability globally (Jat et al., 2021; Yuan et al., 2023b). The worldwide population is exerting a noteworthy burden on land resources due to intensive land cultivation strategies that destroy the soil and environmental quality. Consequently, soil quality must be sustained to certify crop yield and soil and environmental sustainability (Lal, 2005; Pu et al., 2022).

Sustainable crop production in the agricultural industry is highly dependent on the sustainability of the soil system and restricted by soil properties (physical, chemical, and biological) (Wang et al., 2008; Wozniak and Gos, 2014; Indoria et al., 2016). Land management strategies that would fulfill the food demand globally and preserve as well as conserve the previously stressed environmental conditions (Lal, 2005) are significant to sustainable crop production. Conservation tillage practices, for instance, straw-retention or residue mulch and no-tillage systems, are commonly advocated to preserve the soil (Wulanningtyas et al., 2021; Krauss et al., 2022). No-tillage with a crop stubble integration is a more operative approach for enhancing the properties of soil (Yuan et al., 2023a,b), crop yield (Sadiq et al., 2021b), and reduction of global warming (Alhassan et al., 2021), as well as preserving soil health by increasing the quality of soil (Sadiq et al., 2021a). Nevertheless, the influence of conservation tillage practices on soil properties, crop yield, and global warming mitigation has been intensively discussed due to the extensive contradiction in distinct field research (Zheng et al., 2014). Previous studies have depicted that conservation tillage significantly reduced soil properties (Zhao et al., 2014; Khan et al., 2017), crop yield (Taa et al., 2004), and greenhouse gas emissions (Pu et al., 2022; Salamanca-Fresno et al., 2022; Li et al., 2023). On the contrary, conservation tillage pointedly increased soil properties (Jat et al., 2020; Sadiq et al., 2021a), crop yield (Sadiq et al., 2021b; Yuan et al., 2023a), and greenhouse gas emissions (Dencso et al., 2020). Researchers have also demonstrated insignificant change regarding soil properties (Bayer et al., 2015), yield (Lampurlanes et al., 2002), and greenhouse gas fluxes (Bayer et al., 2015) under conservation agriculture and conventional tillage practice. Consequently, more study on the influence of soil tillage on soil properties, crop yield, and global warming is needed.

The accumulation of GHG in the atmosphere alters the earth’s energy balance and participates in the boosted “greenhouse effect.” Additionally, GHG emissions have also been implicated in environmental chemistry given their contribution to the depletion of stratospheric ozone (IPCC, 2013). Global warming is chiefly attributed to the raised GHG concentrations in the atmosphere by anthropogenic activities (O’Neill et al., 2021; Wu et al., 2022). Soil management activities and climatic situations determine the croplands’ capacity to yield, transport, and consume GHG and accordingly determine the direction and intensity of GHG fluxes in farmlands. Conservation tillage practices have been proposed as a substitute land management technique for CT that can mitigate the agricultural sector’s environmental influence through a reduction of GHG emissions (Li et al., 2023). Higher CO_2_ emission was stated by Yeboah et al. (2016) and Dencso et al. (2020) under conservation tillage, while Salamanca-Fresno et al. (2022) reported an almost 50% reduction in CO_2_ flux in conservation tillage treatment compared with CT. In agroecosystems, CH_4_ is also a significant greenhouse gas and acts as a sink or source (Maucieri et al., 2021). The CH_4_ uptake improved under conservation tillage in comparison with CT (Alhassan et al., 2021; Pu et al., 2022); however, Maucieri et al. (2021) did not find any significant difference between conservation and conventional agricultural practices. Regarding N_2_O flux, Yuan et al. (2022) found a reduction in its emission under conservative tillage systems, whereas Pu et al. (2022) reported the maximum emission of N_2_O under conservation agriculture over CT. Moreover, soil physical, chemical, and biological quality indicators greatly influenced GHG fluxes (Alskaf et al., 2021; Hu et al., 2022). A significant correlation between positive and negative GHG fluxes and soil properties was found by many scholars globally (Yeboah et al., 2016; Feng et al., 2018; Huang et al., 2018; Mei et al., 2018; Alhassan et al., 2021; Shakoor et al., 2021; Li et al., 2023). Greater inconsistency in emissions of major GHG from croplands under divergent land management practices has required further investigation under site-specific and soil conditions.

Agroecosystems’ response to diverse soil tillage management systems strongly depends on the local environmental and socioeconomic conditions (Schwilch et al., 2015; Smart SOIL, 2015; Sanz et al., 2017). In this context, broadening the spectrum of scientific research is very essential to cover various environmental (i.e., soil type and climate) and socioeconomic (i.e., funding, market price variations, and type of crop) conditions. In this background, despite the fact that there are numerous studies available worldwide, there is a lack of studies exploring the effectiveness of different conservation tillage practices toward the improvement of soil physical, chemical, and biological properties and crop yield while reducing greenhouse gas emissions and global warming. Too, few studies exist on some selected soil properties and one or two (carbon and nitrous oxide) major greenhouse gas emissions. Studies on greenhouse gas (carbon, methane, and nitrous oxide) emissions, wheat yield, and soil properties (physical, chemical, and biological) responses to different conservation tillage systems on the Loess Plateau, particularly at the Dingxi Belt, are scarce in spring wheat mono-cropping conditions and abundant.

This research tested the hypothesis that conservation tillage practices CTS, NT, and NTS in spring wheat agroecosystems provide better soil physical, chemical, and biological quality indicators and crop yield and yield-attributing traits and reduce the global warming potential of carbon dioxide, methane, and nitrous oxide compared with conventional tillage system. The overall objective of this research was the exploration of soil and environmental quality-based management practices and parameter identification that are sensitive to disturbance of soil. The specific objectives were (i) to evaluate the influence of conservation tillage systems: stubble incorporation with conventional tillage (CTS), no-tillage (NT) and straw-retention with no-tillage (NTS) in the improvement of numerous soil quality indicators, thereby increasing physical, chemical and biological properties; (ii) to assess the effect of the conservation tillage strategies on spring wheat yield and yield-attributing traits; and (iii) to quantify the impact of the conservation tillage techniques on greenhouse gas emissions, namely carbon dioxide, methane, and nitrous oxide emission and also the response of greenhouse gas fluxes to variations in environmental variables because of different tillage systems.

## 2 Materials and methods

### 2.1 Field site description and history of experiment region

**Figure 1:**
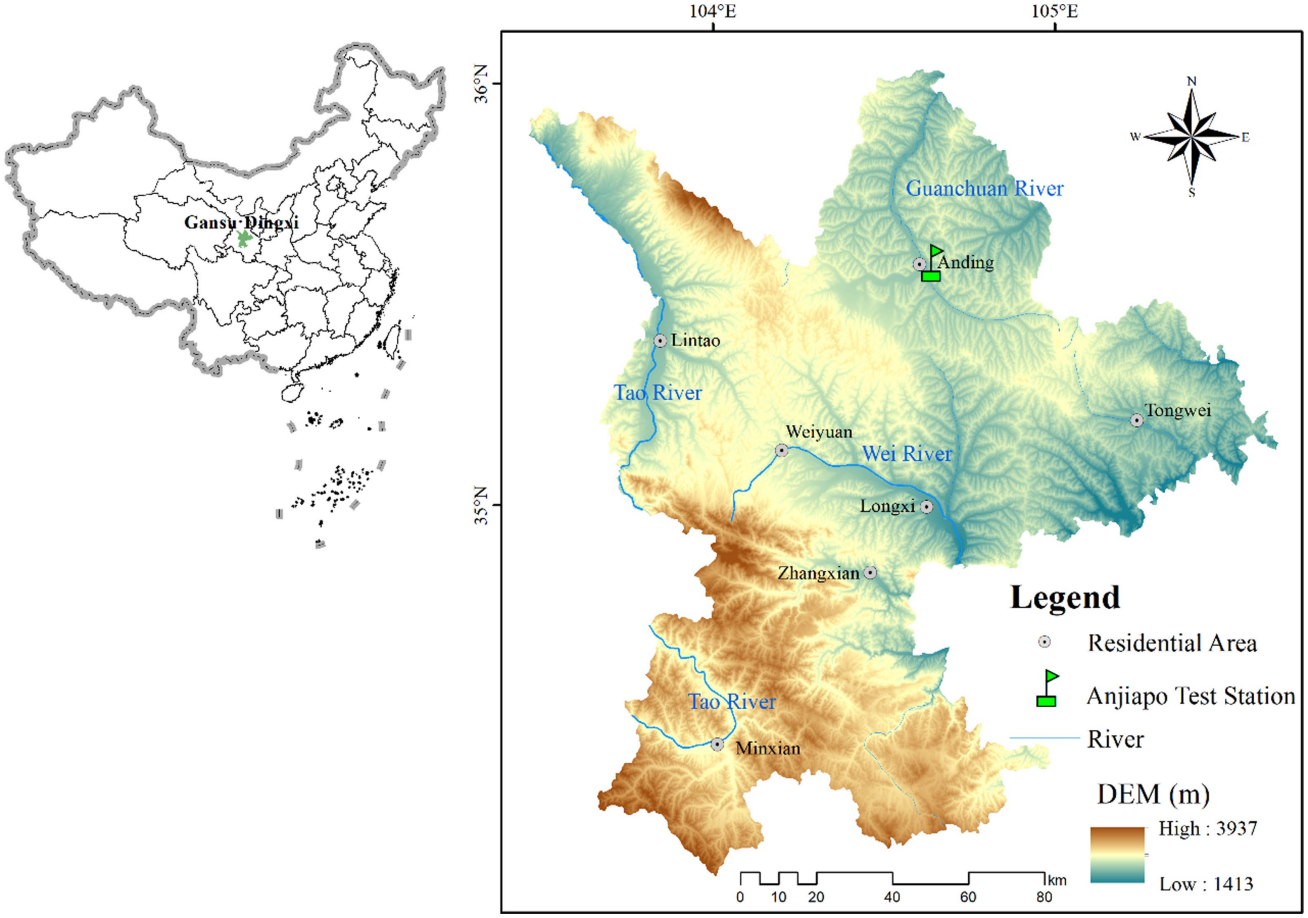
The geographical map of the experimental research site in Dingxi County, Gansu Province, China. ArcGIS 10.2 software was applied for production. The basic geographic information data come from the resource and environmental science and data center (http://www.resdc.cn/).

The field experiment, installed in 2016, is situated on a Calcaric Cambisol at the Anjiapo catchment of Loess Plateau in Dingxi under the Department of Soil and Water Conservation Administration, Gansu Province, Northwestern China (35°34′53′′ N, 104°38′30′′ E), as shown in Figure 1. The study site relief is gently sloped, and the altitude is 2,000 m above sea level. The research county has semi-arid climatic conditions. The type of soil in the study region is a Huangmian sandy loam texture (sand: 60.5%, silt: 24.3%, and clay: 15.2%) according to WRB (IUSS Working Group, 2006), having low soil organic carbon with a slightly alkaline pH (Chinese Soil Taxonomy Cooperative Research Group, 1995).

**Figure 2:**
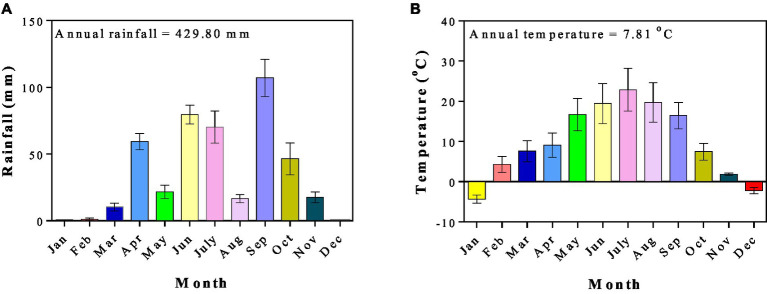
Climatic conditions of the study region in 2021. **(A)** The monthly average rainfall and **(B)** the monthly average temperature.

The average temperatures in this area are −22°C and 35°C in the coolest and warmest months, respectively, and regular frosts in the winter (Xingchen et al., 2019). We have 50 years of continuous climatic data from 1971 to 2020 for this research. The 50-year average annual rainfall from 1971 to 2020 was 400 mm per year, with an irregular distribution. The 50-year average annual radiation is 5,930 MJ m^−2^ with 2,480 h of sunshine per year. This area has 140 days of frost-free period, an average annual evaporation of 1,531 mm, and an annual temperature of 6.9°C from 1971 to 2020. The monthly average of rainfall and temperature for the study period in 2021 is presented in Figure 2.

Before the experiment in 2016, the research field area was bare, which was cut and cleared for spring wheat cultivation. Then, spring wheat was introduced and cultivated at different sowing times (early, normal, and late) under tillage systems. The tillage practices were the same for all spring wheat sowing dates. The Dingxi in Gansu, China, is a research hub and has an extensive wheat cultivation history, and wheat straws were frequently removed prior to the subsequent crop cycle. A comprehensive experiment field site description has been provided in previous studies (Niu et al., 2016; Xu et al., 2020; Sadiq et al., 2021a).

### 2.2 Experimental design

This experimental study was conducted in 2021, from March to August. The research comprised four treatments, including one conventional tillage (CT) control and three different conservation tillage regimes, specifically stubble incorporation with conventional tillage (CTS), no-tillage system (NT), and straw-return with no-tillage (NTS). The conservation tillage regimes were compared with conventional tillage control. The experimental treatments were replicated three times in an 8 m × 3 m plot size under randomized complete block design (RCBD), giving a total of 12 individual plots with a total area of 24 m^2^. These conservation tillage regimes have been practiced since 2016, with 5 years of non-stop spring wheat cultivation. At the beginning of the research, soil samples contained an average bulk density (BD) of 1.41 ± 0.02 g cm^−3^, soil porosity (P) 47.05 ± 3.31%, total nitrogen (TN) 0.59 ± 0.02 g kg^−1^, total phosphorous (TP) 0.43 ± 0.03 g kg^−1^, total potassium (TK) 18.48 ± 0.01 g kg^−1^, soil organic carbon (SOC) 5.85 ± 0.34 g kg^−1^, and pH 8.34 ± 0.05 in 0–15 cm depth (Table 1).

**Table 1:** The basic characteristics of 0–15 cm soil depth at the Dingxi research field in 2021.

Soil parameter	Values	Measurement method	References
BD (g cm^−3^)	1.41 ± 0.02	Core sampler method	Lu (1999)
P (%)	47.05 ± 3.31	(1 − (BD/PD)) × 100 equation	Lu (1999)
TN (g kg^−1^)	0.59 ± 0.02	Semimicro-Kjeldahl method	Lu (1999)
TP (g kg^−1^)	0.43 ± 0.03	Colorimetric method	Lu (1999)
TK (g kg^−1^)	18.48 ± 0.01	Colorimetric method	Lu (1999)
SOC (g kg^−1^)	5.85 ± 0.34	Walkley-Black dichromate oxidation	Nelson and Sommers (1983)
pH	8.34 ± 0.05	pH meter	Lu (1999)
Soil textural class	Sandy-loam	Hydrometer method	Bouyoucos (1927)

The abbreviated words stand for BD, bulk density; P, soil porosity; TN, total nitrogen; TP, total phosphorous; TK, total potassium; SOC, soil organic carbon; pH, soil pH; PD, particle density = (2.65 g cm^−3^). Based on USDA soil textural classification.

### 2.3 Tillage systems and crop management practices

The soil tillage management practices compared in this trial were conventional tillage (CT), stubble incorporation with conventionally tilled soil (CTS), no-tillage (NT), and no-till with straw-retention (NTS). Descriptions of these tillage treatments are presented in Table 2. For the CT system, land cultivation was performed three times with moldboard plow in a year at 20 cm, 10 cm, and 5 cm, respectively, and harrowed two times, followed by planting in the absence of stubble. In order to manage CTS, after harvesting the wheat crop, the fields were plowed with moldboard plow, harrowed, and followed by planting exactly as for the CT practice (three plow passes and harrows two times), but stubble incorporation was done at the time of first plowing. After threshing, all the wheat-straw from the earlier spring wheat crop was returned to the original plot instantly and then incorporated into the field. The spring wheat was planted exactly as in the CT system. In the NT-treated plots, after harvesting the spring wheat, all stubbles were removed and crop planting was performed with a no-tillage crop planter; nevertheless, in the NTS practice, after harvesting the wheat, all stubble was returned to the field and crop sowing was done with a no-tillage crop planter. The wheat-stubble chemical composition is shown in Table 3. The wheat-straw nitrogen, phosphorous, potassium, and carbon were 0.79 ± 0.2%, 0.08 ± 0.01%, 0.49 ± 0.04%, and 39.24 ± 2.8%, respectively. Nitrogen at the rate of (146 kg ha^−1^) and phosphorous at the rate of (63 kg ha^−1^) were applied as basal doses in all treatments, counting control in the form of diammonium phosphate and urea. Semi-arid Dingxi in the Loess Plateau zone of China has a suitable concentration of soil potassium (Li et al., 2014), which was satisfactory for encouraging wheat growth and germination; therefore, potassium was not applied in basal fertilization. The spring wheat (cultivar Dingxi 42) crop was sown by hand in mid-March (seed rate was 187.5 kg seeds ha^−1^) and allowed to grow until late-August (Table 3). The wheat crop was sown using 20 cm row-to-row spacing and a plant density of 400 plant m^−2^. In order to control the weeds, the red sun herbicide with glyphosate (30%) was used according to the manufacturer’s instructions, and when weeding was required during the growing season, it was done manually.

**Table 2:** Treatment details of tillage regimes and stubble application for spring wheat cultivation tested during the course of this study.

Treatments	Short forms	Description
Conventional tillage + straw harvest	CT	After harvesting the wheat crop, the fields were plowed three times with moldboard plow and harrowed two times followed by planting. The first plowing was done in late-August immediately after harvesting the spring wheat crop, in late-August and late-September, the second and third plowing, respectively, were done. The depths of plow were 20 cm, 10 cm, and 5 cm, respectively. The field harrowing was done before the ground was frozen. This is the typical conventional tillage technique in the Dingxi zone of China. The spring wheat crop was planted with a small seeder drawn by a 13.4 kW (18 HP) tractor and designed by China Agricultural University, letting fertilizers be positioned under the seed rows, followed by concave rubber press wheels in one operation.
Conventional tillage + stubble incorporation	CTS	After harvesting the spring wheat crop, the fields were plowed with moldboard plow, harrowed, and followed by planting exactly as for the conventional tillage practice (3 plow passes and harrows two times) described above, but with stubble incorporated at the time of first plowing. After threshing, all the wheat stubble from the preceding spring wheat crop was returned to the original plot immediately and then incorporated into the field. The spring wheat crop was planted exactly as for the conventional tillage practice.
No-tillage + straw harvest	NT	No-tillage all over the life of the experimental research. The crop stubble was removed from the field and used as feed or fuel. Direct planting with no-till crop planter into 20 cm depth without using any tillage implement.
No-tillage + stubble return	NTS	No-tillage during the experiment life. The field was shielded with the earlier spring wheat crop straw from late-August till the following mid-March. After threshing, all the crop stubble from the previous wheat crop was returned to the original plot immediately. Direct sowing with no-till crop planter into 20 cm deep in the absence of any prior tillage, understanding earlier wheat crop stocks.

CT, conventional tillage is the control for a given study period.

**Table 3:** Wheat-straw properties and crop management practices during the study.

Wheat crop stubble properties						
Nitrogen		Phosphorus		Potassium		Carbon
(%)						
0.79 ± 0.2		0.08 ± 0.01		0.49 ± 0.04		39.24 ± 2.8
Crop management practices during research						
Crop	Crop variety	Plot size	Seed rate (kg ha^−1^)	Plant density (plant m^−2^)	Fertilizer	Weed control (L ha^−1^)
Spring wheat	Dingxi 42	24 m^2^	187.5	400	Diammonium phosphate (146 kg ha^−1^), urea (63 kg ha^−1^)	Herbicide (Red sun) with 30% glyphosate

Spring wheat crop was sown in mid-March and harvested in late-August during the study.

### 2.4 Agronomic traits

The agronomic attributes of plant height, spike number per plant, seed m^2^, root yield, aboveground biomass yield, grain yield, 1,000-grain weights, dry matter yield, and harvest index were determined. The wheat plant height was measured using the procedure described by Dokuyucu et al. (2002) and Demuner-Molina et al. (2014).

### 2.5 Soil measurements

At the spring wheat harvest stage in 2021, five disturbed and undisturbed soil samples were collected with an auger having a diameter of 4 cm from different experimental treatments (CT, CTS, NT, and NTS), including three replications for the determination of soil physicochemical, biochemical, and biological properties. All soil quality indicators were observed at a soil layer of 0–10 cm depth.

The oven-dry method was used for the determination of gravimetric soil water content (SWC) (O’Kelly, 2004), water-filled pore space (WFPS) in percent, and soil water storage (SWS), which was calculated as described by O’Kelly (2004). Soil temperature (ST) was measured using a geothermometer (Lu, 1999). The procedure described by Campbell (1994) was used for soil bulk density (BD) determination with the core sampler method. and soil pore space (P) percent was calculated in accordance with the procedure described by Campbell (1994). The wet-sieved method was used for measurements of soil aggregates (Yang et al., 2018). Auger-hole method using the Guelph Permeameter was used for the determination of saturated soil hydraulic conductivity (Ks) (Reynolds et al., 2008).

The soil organic carbon (SOC) was determined by the standard method of Walkley-Black dichromate oxidation (Nelson and Sommers, 1983), while the density fractionation approach was used for the isolation of light fraction organic carbon (Gregorich and Ellert, 1993), and C and N analyzer (Elementar Vario MACRO cube) was used for its determination. The calculation of soil organic carbon storage (t hm^−2^) was done using the procedure described by Wu et al. (2021). The estimation of microbial biomass carbon (MBC) and microbial biomass nitrogen (MBN) was done by the fumigation-extraction method (Vance et al., 1987).

Soil total nitrogen (TN), total phosphorous (TP), total potassium (TK), available nitrogen (AN), available phosphorous (AP), available potassium (AK), electrical conductivity (ECe), and pH were determined by using standard procedures (Lu, 1999). Nitrogen storage was calculated, followed by the equation of Wu et al. (2021). Soil microbial propagules (colony forming units, CFUs) were determined using the enumeration of luminescent colonies on agar media (Li et al., 1996). Soil urease activity was estimated following the procedures by Dick and Burns (2011), soil alkaline phosphates were estimated as described by Zhao and Jiang (1986), soil invertase activity was determined by the method of Frankeberger and Johanson (1983), soil cellulase activity was estimated following the method of Guan (1986), and soil catalase activity was determined as described by Yan (1988).

### 2.6 Gas sampling and flux measurement

The procedure of greenhouse gas sampling, such as CO_2_, CH_4_, and N_2_O, was conducted during different crop growth stages of spring wheat in 2021. On the basis of the static dark chamber and gas chromatography method described by Yuesi and Yinghong (2003), gas sampling and flux measurements were done. A stainless-steel base with a collar (50 × 50 × 10 cm) was installed to support sampling chamber placement (50 × 50 × 50 cm) for greenhouse gas sampling in each plot (a total of 12 plots). The samples of air were drawn from the chambers simultaneously, including three replications of each treatment. At five different times (0, 9, 18, 27, and 36 min), respectively, by using 150 mL gas-tight polypropylene syringes, the air samples were drawn and released into 100 mL aluminum foil sampling bags (Shanghai Sunrise Instrument Co. Ltd., Shanghai). Then the samples of gas were analyzed in the laboratory with a GC system (Echrom GC A90, China) equipped with a flame ionization detector for methane and carbon dioxide and electron capture detector for nitrous oxide analysis. At 250°C temperature and 35 cm^3^ min^−1^ H_2_ flow rate, the flame ionization detector operates. Methane, carbon dioxide, and nitrous oxide peak areas were analyzed in the Echrom-ChemLab software. Calibrations were done with standard gas obtained from Shanghai Jiliang Standard Reference Gases Co., Ltd., China, before the sample gas analyses. Standard gas concentrations were 2.00 ppmv, 456.00 ppmv, and 0.355 ppmv for methane, carbon dioxide, and nitrous oxide, respectively. In order to obtain the concentration change over sampling time, the concentrations of the sample gas obtained for the five sampling times were plotted against time. Emissions of carbon dioxide in terms of ecosystem respiration, methane, and nitrous oxide fluxes were calculated as described by Wei et al. (2014), followed by equation (1):

(1) $$F=\frac{dC}{dt}\cdot \frac{M}{V_{0}}\cdot \frac{P}{P_{0}}\cdot \frac{T}{T_{0}}\cdot H$$

where *dC/dt* is the rate of gas concentration change; *M* is the molar mass of nitrogen or carbon (28 for N_2_O and 12 for CO_2_ and CH_4_); *V*_o_ is standard molar air volume (22.41 mol^−1^), *P* is the sampling site air pressure; *P*_o_ is the standard air pressure, *T* is the chamber air temperature at the sampling time, *T*_o_ is the standard air temperature; and *H* is the height of the chamber. The cumulative flux of methane, carbon dioxide, and nitrous oxide in kg ha^−1^ was estimated using the following equation (2):

(2) $$M=\sum \frac{\left(F_{i+1}+F_{i}\right)\times \left(t_{i+1}-t_{i}\right)\times 24}{2\times 100}$$

where *M* is the gas cumulative emission during the whole growth period of spring wheat (kg ha^−1^); *F* is the gas emission flux (mg/m^−2^h^−1^); *i* is the sampling number; *t* is the sampling time (d).

In order to estimate global warming potential (GWP) of greenhouse gases, the cumulative flux of CO_2_ in µmol m^−2^s^−1^ was converted to mg CO_2_–C and typically taken as the reference gas; therefore, methane and nitrous oxide emissions are converted into “CO_2_ equivalents” (CO_2_-e). The methane GWP is 34 (based on a 100-year time horizon), the carbon dioxide GWP is 1, and the nitrous oxide GWP is 298 (IPCC, 2013). Net global warming potential (GWP) in kg CO_2_-eq ha^−1^ was determined by using equation (3):

(3) $$\begin{array}{l} Net\;GWP={CH}_{4}\;\mathrm{flux}\times 34+N_{2}O\;\mathrm{flux}\\ \;\times 298+Net\;CO_{2}\;\mathrm{flux} \end{array}$$

Greenhouse gas intensity (GHGI) was determined by using equation (4):

(4) $$\mathrm{GHGI}=\frac{GWP}{\mathrm{Grain}\;\mathrm{yield}}$$

The sign convention adopted is that positive (+) means emission, whereas negative (−) means absorption.

### 2.7 Statistical analysis

The experimental data were processed using Excel 2016 (Microsoft Corp., United States). The procedure used to analyze the data obtained from the experiment was one-way factor interaction analysis of variance (ANOVA), suitable for randomized complete block design (RCBD). The linear model procedure of the appropriate computer software program Statistical Package for Social Science (SPSS) window version 25.0 (IBM Corp., Chicago, IL, USA) was used for all statistical analysis. The significant differences among different treatments were separated by Duncan’s test at 5% significance level (*p* < 0.05). Data obtained from treatments are displayed as the mean of three replications with standard deviation and computer software Origin 2021 was used for drawing figures. Additionally, in order to explore the multivariate variability introduced by the various tillage practices for soil properties, crop yield, and greenhouse gases, the principle component analysis (PCA) was performed. The Pearson heatmap correlation analysis was used to describe the relationship among the factors.

## 3 Results and discussion

### 3.1 Effect of conservation tillage on wheat agronomic traits

Conservation tillage management had an influence on spring wheat agronomic features, including growth, yield, and yield-attributing traits. The NTS practice generated maximum 95.26 ± 3.08 cm plant height, 44.66 ± 5.12 number of spikes per plant, seeds number per meter square 9321.97 ± 623, root yield (RY) 532.88 ± 24 kg ha^−1^, aboveground biomass yield (ABY) of 5225.31 ± 327 kg ha^−1^, thousand-grain weight (TSW) 50.95 ± 2.02 g, grain productivity (GY) of 2543.88 ± 275 kg ha^−1^ and 2681.43 ± 257 kg ha^−1^ dry matter yield (DMY) while lowest values of all investigated agronomic characters were associated with CT technique. The agronomic parameters of wheat followed the trend of CT > NT > CTS > NTS, except for plant height and spike number per plant, where no-till depicted better performance over CTS. However, CT produced pointedly highest 48.76 ± 1.33% harvest index (HI) compared with other tested conservative tillage treatments (Table 4). This research verified our hypothesis that a conservation tillage technique can improve productivity; it would be a sustainable and reliable agronomical practice in dry regions.

**Table 4:** Wheat agronomic traits as influenced by conservation tillage practices in 2021.

Spring wheat agronomic attributes									
Treatment	Plant height (cm)	Number of spikes plant^−1^	Seed (n m^−2^)	Root yield (kg ha^−1^)	Aboveground biomass yield (kg ha^−1^)	Thousand seed weight (g)	Grain yield (kg ha^−1^)	Dry matter yield (kg ha^−1^)	Harvest index (%)
CT	81.16 ± 2.05b	31.66 ± 4.04b	7463.14 ± 498b	476.62 ± 13b	3412.74 ± 130b	44.52 ± 1.05b	2200.10 ± 123b	1193.64 ± 211b	65.23 ± 2.36a
CTS	90.02 ± 3.15ab	34.33 ± 5.04ab	9175.63 ± 545a	520.45 ± 22a	4223.95 ± 363ab	49.58 ± 2.34a	2277.62 ± 232ab	1946.33 ± 230ab	54.25 ± 2.12ab
NT	92.23 ± 2.52ab	36.66 ± 3.74ab	8902.46 ± 474ab	485.76 ± 16ab	3786.04 ± 284ab	45.95 ± 1.65ab	2219.92 ± 214ab	1578.11 ± 248ab	58.31 ± 1.45ab
NTS	95.26 ± 3.08a	44.66 ± 5.12a	9321.97 ± 623a	532.88 ± 24a	5225.31 ± 327a	50.95 ± 2.02a	2543.88 ± 275a	2681.43 ± 257a	48.76 ± 1.33b
*p*-value	0.024	0.041	0.027	0.033	0.012	0.048	0.039	0.016	0.046

Mean values with the same letter in a column are not significantly different between each treatment at *p* < 0.05 (Duncan’s test was performed for mean separation). Values are means ± SE (*n* = 3). CT: conventional till; CTS: conventional till with stubble incorporation; NT: no-till; NTS: no-till with stubble return. *p*-value in a column indicates the level of probability among treatments.

Soil inversion elimination and crop stubble implementation are the most significant practices in the agriculture production system. The data reveal that different conservation tillage systems are suitable for producing spring wheat in the semi-arid Dingxi belt. The significant maximum agronomic trait values of conservative tillage systems indicate the competent use of stubbles (Zhao et al., 2019; Jat et al., 2021; Li et al., 2023), water, and nutrients. The better wheat agronomic performance was due to straw-retention because crop stubble has the potential to retain essential soil nutrients, which led to increased crop agronomic parameters (Han et al., 2020). This is consistent with previous results (Sadiq et al., 2021b), in which they recorded increased crop yield under conservation agricultural practices and associated it with residue retention. The higher wheat yield and yield-attributing traits under NTS, CTS, and NT systems than CT can further be ascribed to increased soil organic matter, which improves soil quality and leads to increased crop agronomic parameters (Sadiq et al., 2021a). Maximum crop production under straw-return and residue incorporation and the NT system over CT might also be due to better hydrothermal soil characteristics (Huang et al., 2012; Wang et al., 2017). Our current study results are in agreement with the findings of Alhassan et al. (2021), as they verified that stubble return and residue incorporation and no-till systems enhanced wheat yield compared with CT in a dry region farming system.

### 3.2 Soil properties as influenced by conservation tillage management practices

**Figure 3:**
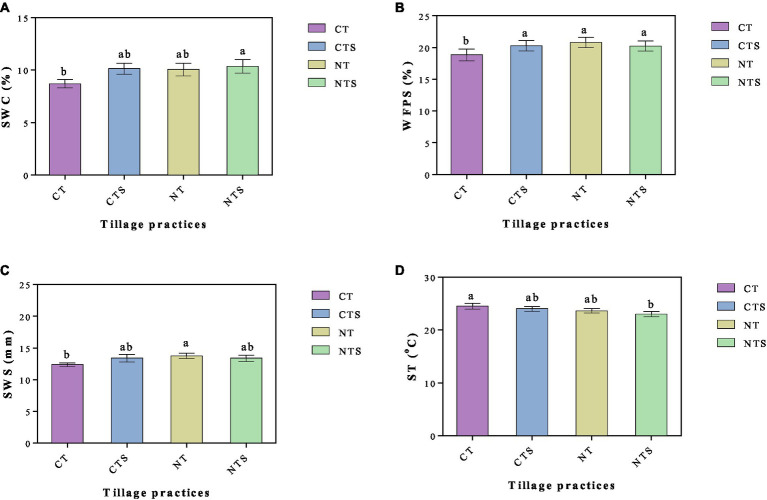
Properties of soil under conservative tillage systems in 2021 at 0–10 cm soil depth. **(A)** The soil gravimetric water content influenced by conservation tillage technique; **(B)** the water-filled pore spaces under conservation tillage; **(C)** the soil water storage affected by tillage practices; and **(D)** the soil temperature affected by tillage measures. Vertical error bars denote the corresponding standard error of mean values; n = 3. Significant differences were determined by a Duncan’s test.

Conservation tillage systems have noteworthy impact on soil properties, for instance, soil water content (SWC), water-filled pore space (WFPS), soil water storage (SWS), soil temperature (ST), soil bulk density (BD), soil porosity (P), aggregates, soil saturated hydraulic conductivity (Ks), soil organic carbon (SOC), light fraction organic carbon (LFOC), carbon storage (CS), microbial biomass carbon (MBC), total nitrogen (TN), available nitrogen (AN), nitrogen storage (NS), microbial biomass nitrogen (MBN), total phosphorous (TP), available phosphorous (AP), total potassium (TK), available potassium (AK), microbial counts (MC), urease, alkaline phosphatase, invertase, cellulase, and catalase at a soil depth of 0–10 cm (Figure 3 and Table 5). The concentration of SWC (*p* < 0.05) was maximum (10.35 ± 0.64%) in the NTS treatment variant with two other CTS and NT conservative tillage treatments, while CT had a minimum (8.70 ± 0.40%) SWC value. The conservation tillage practices NTS, CTS, and NT increased SWC by 9.35, 9.13, and 9.05%, respectively, over the CT system. The WFPS was highest (20.80 ± 0.81%) under the NT system, which was followed by NTS and CTS techniques, while the lowest WFPS was associated with CT practice. Additionally, the same trend was observed in the case of SWS, with the highest value in NT and the lowest in CT. The rise in SWC, WFPS, and SWS under CTS and NTS measures may be owing to crop straw-retention, thus decreasing evaporation losses, improving water availability and infiltration rate, and conserving soil water (Yadav et al., 2018; Zhang et al., 2018). Additionally, crop stubble addition to soil increases SOM and thus improves soil water retention capacity. The increase in SWC, WFPS, and SWS under NT practice over the CT system might be due to the earth flip inherent to CT practice, resulting in a big water loss due to evaporation. This water loss is avoided in NT, which lacks the requirement for stubble addition (Liebhard et al., 2022). Parallel findings were found by Yuan et al. (2023a).

**Table 5:** Changes in soil properties under sustainable conservation tillage in 2021 at 0–10 cm soil depth.

Treatment	BD (g cm^−3^)	P	Aggregates (%)	Ks (mm h^−1^)	SOC (g kg^−1^)	LFOC (g kg^−1^)	Carbon storage (t hm^−2^)	MBC (mg kg^−1^ soil)	TN (g kg^−1^)
CT	1.42 ± 0.03a	46.28 ± 1.34b	13.41 ± 1.22b	0.92 ± 0.06b	9.86 ± 0.3b	0.86 ± 0.04b	12.57 ± 0.51b	153.33 ± 14.50b	0.61 ± 0.02b
CTS	1.33 ± 0.05ab	50.06 ± 1.63a	18.13 ± 1.02ab	1.36 ± 0.32ab	11.22 ± 0.7ab	1.38 ± 0.08ab	14.81 ± 0.90ab	247.00 ± 15.56a	0.74 ± 0.03a
NT	1.37 ± 0.05ab	48.17 ± 0.84ab	16.59 ± 1.50ab	1.84 ± 0.10a	11.08 ± 0.6ab	1.08 ± 0.07ab	15.23 ± 1.16a	228.00 ± 12.28ab	0.69 ± 0.05ab
NTS	1.29 ± 0.04b	51.19 ± 1.27a	20.18 ± 2.44a	1.95 ± 0.05a	11.87 ± 0.5a	1.49 ± 0.09a	15.36 ± 0.63a	243.64 ± 7.23a	0.68 ± 0.06ab
*p*-value	0.025	0.037	0.022	0.035	0.034	0.024	0.041	0.012	0.021
	AN (mg kg^−1^)	Nitrogen storage (t hm^−2^)	MBN (mg kg^−1^ soil)	TP (g kg^−1^)	AP (mg kg^−1^)	TK (g kg^−1^)	AK (mg kg^−1^)	ECe (dSm^−1^)	pH
CT	40.24 ± 1.64b	0.78 ± 0.05b	37 ± 3.5b	0.40 ± 0.03b	13.74 ± 1.63b	17.67 ± 0.01b	221.33 ± 6.02b	0.37 ± 0.01a	8.40 ± 0.02a
CTS	47.40 ± 2.18a	0.92 ± 0.07a	52 ± 6.60a	0.50 ± 0.06a	18.64 ± 2.54ab	18.84 ± 0.02a	272.91 ± 8.12ab	0.41 ± 0.02a	8.37 ± 0.05a
NT	43.21 ± 2.13ab	0.94 ± 0.02a	43 ± 7.21ab	0.47 ± 0.08ab	17.41 ± 1.92ab	18.72 ± 0.03ab	253.84 ± 7.48ab	0.39 ± 0.02a	8.38 ± 0.053a
NTS	46.47 ± 2.26a	0.86 ± 0.04ab	53 ± 6.29a	0.44 ± 0.07ab	19.42 ± 2.39a	18.90 ± 0.02a	292.83 ± 9.84a	0.40 ± 0.03a	8.36 ± 0.06a
*p*-value	0.030	0.027	0.019	0.045	0.016	0.048	0.018	0.054	0.058
	C:N ratio	C:P ratio	N:P ratio	Microbial counts (10^4^ CFU g^−1^ soil)	Urease (mg [NH_3_–N] g^−1^ soil d^−1^)	Alkaline phosphatase (mg [phenol] g^−1^ soil d^−1^)	Invertase (mg [glucose] g^−1^ soil d^−1^)	Cellulase (mg [glucose] g^−1^ soil d^−1^)	Catalase (ml [0.1 mol L^−1^ KMnO_4_] g^−1^ soil h^−1^)
CT	16.46 ± 0.77a	26.47 ± 2.10a	1.48 ± 0.19a	111.66 ± 9.52cb	2.47 ± 0.04b	0.75 ± 0.03b	17.56 ± 1.5b	5.53 ± 1.04b	5.09 ± 0.03b
CTS	16.06 ± 1.81a	21.03 ± 2.12a	1.40 ± 0.15a	131.33 ± 17.12ab	2.58 ± 0.09ab	0.85 ± 0.06ab	19.33 ± 2.2ab	7.41 ± 1.72ab	5.20 ± 0.07ab
NT	16.19 ± 1.74a	21.14 ± 4.16a	1.49 ± 0.23a	136.33 ± 12.25ab	2.59 ± 0.08ab	0.90 ± 0.05ab	20.52 ± 1.7ab	7.91 ± 1.24ab	5.22 ± 0.04ab
NTS	17.75 ± 1.02a	19.78 ± 2.63a	1.50 ± 0.10a	146.66 ± 15.41a	2.64 ± 0.07a	0.97 ± 0.04a	23.8 ± 2.4a	9.11 ± 0.98a	5.36 ± 0.06a
*p*-value	0.062	0.053	0.066	0.022	0.044	0.033	0.039	0.043	0.046

Mean values with the same letter in a column are not significantly different between each treatment at *p* < 0.05 (Duncan’s test was performed for mean separation). Values are means ± SE (*n* = 3). CT: conventional till; CTS: conventional till with stubble incorporation; NT: no-till; NTS: no-till with stubble return. *p*-value in a column indicates the level of probability among treatments.

The CT had the highest ST, whereas the NTS had the minimum ST values. The temperature moderation influence was observed in conservative tillage treatments as decreased ST by NTS, NT, and CTS systems (Figure 3). This is due to the accumulation of crop straws. The surface of the soil is abstemiously shielded by stubble remnants from prior crops in conservative tillage systems, encouraging the soil to absorb minimum solar radiation (Wang et al., 2009). Furthermore, the CT practice makes the soil more porous, and therefore, the soil under CT possibly has minor thermal conductivity (Sarkar and Singh, 2007). In addition, conservative tillage treatments increased the infiltration rate, which helped water movement toward the bottom (Liebhard et al., 2022) and reduced ST (Yuan et al., 2023a). Similar observations were noted by Sadiq et al. (2021a).

On average, conservation tillage systems NTS, CTS, and NT reduced BD by 29, 33, and 37%, respectively, over CT. The maximum soil BD under CT was connected to the soil disturbance by tillage implementation, which resulted in soil compactness (Gathala et al., 2011). In addition, due to traffic physical compaction by direct heavy machinery (Jat et al., 2018). In addition, as BD values are connected with soil porosity values, these conservative tillage systems improved the *p*-values, with the highest under NTS and the lowest recorded in CT. The NTS, CTS, and NT increased the soil porosity because of the organic matter accumulation under conservation tillage, which led to decreased BD and improved *p*-values (Ordoñez-Morales et al., 2019). Similar results were noted by Khorami et al. (2018), who recorded that conservation tillage decreased the BD and increased pore space over CT.

Almost similar to SWC, NTS attained the highest soil aggregates, followed by CTS, which was statistically on par with NT, while CT linked with the lowest soil aggregates. The CT strongly disturbs the soil due to plowing, which can diminish the aggregate degree and aggregate stability (Yeboah et al., 2018). Higher aggregates in conservation tillage might be due to residue retention (Niu et al., 2016). Similar results are reported in accordance with (Yuan et al., 2023a). Additionally, conservation tillage practices increased the Ks over CT; the highest value of Ks was reported in NTS, which was statistically at par with NT, followed by CTS, while the lowest Ks value was associated with CT. The minimum soil Ks in CT and CTS might be ascribed to the destruction of soil aggregation and reduction of macro-porosity (Singh et al., 2002). In arid and semi-arid cropping systems, the soil property variations under conservation tillage influences are habitually slow to arise, attributable to the limited production of plant biomass (Mikha et al., 2006).

The SOC, LFOC, CS, and MBC differed significantly (*p* < 0.05) in all tested tillage systems, whereas the non-significant (*p* < 0.05) differences were noted among CTS and NT for SOC and LFOC as well as NT and NTS had also non-significant differences for carbon storage, and in case of MBC, the NTS and CTS are statistically at par (Table 5). The NTS system had the highest values of SOC, LFOC, and carbon storage except for MBC, where CTS was related to higher values, while the CT system had the lowest values. The SOC, LFOC, carbon storage, and MBC contents were low in the CT system because of extensive tillage operations, breakdown of aggregates, and exposure of the soil carbon contents. More values of carbon-associated parameters in CTS, NTS, and NT systems soil were attributable to residue retention and no soil physical disturbance (Chen et al., 2009). In conservative tillage systems, the SOC solid stratification, which was significantly attributable to surface residue retention, is identical to that in other studies (Blanco-Canqui and Lal, 2008; Yuan et al., 2023b). Soil carbon storage is the retention of carbon in the ecosystem and a chief index to measure the gauge and quantity of ecosystem primary productivity. A study by Yeboah et al. (2018) revealed that NTS can significantly increase the soil organic carbon content so as to progress soil carbon storage. This study also found that compared with CT, conservation tillage systems were more helpful in improving soil carbon storage. The higher values of MBC appear because of high microbial biomass and soil organic matter contents. Organic stubbles are used as carbon source inputs for the microorganisms’ activities, activation with the support of microbial biomass, conservation tillage systems enrich the soil carbon contents (Liu et al., 2017). The role of straw-retention and residue incorporation in increasing carbon elements was also stated by numerous scholars (Zhao et al., 2019; Sadiq et al., 2021a; Yuan et al., 2023b). The conservation tillage system is a straw addition or plowless tillage with a minimum number of tillage operations, and its beneficial impact on soil functions and quality has been extensively identified (Han et al., 2020; Wulanningtyas et al., 2021; Sadiq et al., 2021b).

Conservation tillage practices (NTS, CTS, and NT) significantly improved total and available soil nutrients, namely TN, AN, TP, AP, TK, and AK, compared to CT. In addition, NC and MBN were notably increased under these investigated conservative tillage systems over CT. Higher TN, AN nitrogen storage, and TP were related to CTS but the NTS system had dominant MBN, AP, TK, and AK values, while CT had the lowest values. Beneficial influence of NTS, CTS, and NT on nutrient accumulation and storage might be due to multiple reasons:

- Higher total and available soil nutrients, for instance, nitrogen, phosphorous, and potassium in CTS and NTS, might be because of straw-retention because straw has the potential to add essential nutrients to the soil system (Zhao et al., 2019).
- Maximum nitrogen accumulation under straw-treated treatments may be attributed to straw microbial biomass and nitrogen immobilization (Huang et al., 2012).
- Stubble retention and incorporation can decrease leaching and volatilization losses of nitrogen by diminishing the soil temperature and lead to increased nitrogen accumulation (Sadiq et al., 2021a).
- Highest nitrogen accumulation under conservative tillage treatments (CTS, NT, and NTS) may also be due to better biological activity (Wang et al., 2017).
- Straw can increase organic matter and lead to improved phosphorous solubilization and fixed potassium availability by reducing phosphorous adsorption to mineral surfaces (Celik et al., 2011) and potassium adsorption to clay mineral surfaces (Celik et al., 2011).

Our findings concur with those of Zhao et al. (2019), Han et al. (2020), Sadiq et al. (2021a), Wulanningtyas et al. (2021), Lv et al. (2023), who found that nutrient elements and their storages were higher under conservation tillage than plowed ones.

The ECe, pH, C:N ratio, C:P ratio, and N:P ratio under different conservation tillage measures were inconsistent and non-significant. However, CTS, NT, and NTS showed a trend of increased ECe and decreased pH compared with CT. The dropping of pH under conservative tillage was previously described by numerous scholars (Jat et al., 2018; Sinha et al., 2019). Soil pH reduction in CTS, NT, and NTS conservation-based tillage regimes may be owed to organic acid production during retained stubble decomposition (Sinha et al., 2019). The C:N ratio was lower in CTS and NT than in CT, which might be due to slower decomposition under these systems. Our results are in line with those (Jat et al., 2018), which pointed out the minor values of C:N ratio under conservative tillage over CT. In general, the soil C:P and N:P ratios were highest under CT and NTS treatment, respectively. These results are in agreement with Feng et al. (2014), who stated that NT practice coupled with straw increases the soil N:P ratio.

Conservation tillage system had a great influence on soil microbial count and enzymatic activities. The highest values of soil microbial count, urease, alkaline phosphatase, invertase, cellulase, and catalase were obtained under NTS treatment, followed by CTS and NT, while the CT system had the lowest values of all these parameters. The conservative tillage systems NTS, CTS, and NT increased microbial count by 31.34, 17.61, and 21.19%, respectively; urease activity by 6.88, 4.45, and 4.85%, respectively; activity of alkaline phosphatase by 29.33, 13.35, and 20%, respectively; invertase activity by 35.53, 10.07, and 16.85%, respectively; cellulase activity by 64.73, 33.99, and 43.03%, respectively; and catalase activity by 5.30, 2.16, and 2.55%, respectively, over CT. The microbial count and enzyme activity depicted the trend of CT < CTS < NT < NTS. The soil microorganisms are a significant portion of the cropland ecosystem and are involved in the decomposition of SOM, nutrient circulation in the soil system, humus formation, and soil fertility (Yu, 2015). Soil microbes’ stability is a vital indicator of soil quality, health, and fertility (Yang et al., 2023). Land cultivation methods and cropland management practices affect the soil microbial community. Conservation tillage has the potential to improve the soil microbial community and the activities of soil enzymes (Rakesh et al., 2021). This study depicts that NTS, CTS, and NT raised the microbial community and activities of urease, alkaline phosphatase, catalase, invertase, and cellulase in the soil system to a greater extent than the CT system. This shows that conservative tillage mechanically makes the soil more porous (Yang et al., 2018) and increases the SOM distribution while facilitating the activity of enzymes (Balota et al., 2004). Hence, stubbles coupled with no-tillage (NTS) improved the soil microbial count, urease, alkaline phosphatase, invertase, cellulase, and catalase, which improved crop germination, growth, and yield. Optimal soil physicochemical properties could increase enzyme activity (Wei et al., 2013). In this context, NTS, CTS, and NT treatments were more effective than CT control treatment. Measurements of soil characteristics and variations are crucial for evaluating the influence of soil management techniques (Jat et al., 2021). The improved enzyme activities may be because of augmented carbon sequestration and immobilization of carbon as well as nitrogen during the decomposition of residues, as numerous studies have revealed that the activities of soil enzymes can be influenced by SOC sequestration (He et al., 2020; Pu et al., 2020). Our results are in accordance with Bhattacharya et al. (2020).

**Figure 4:**
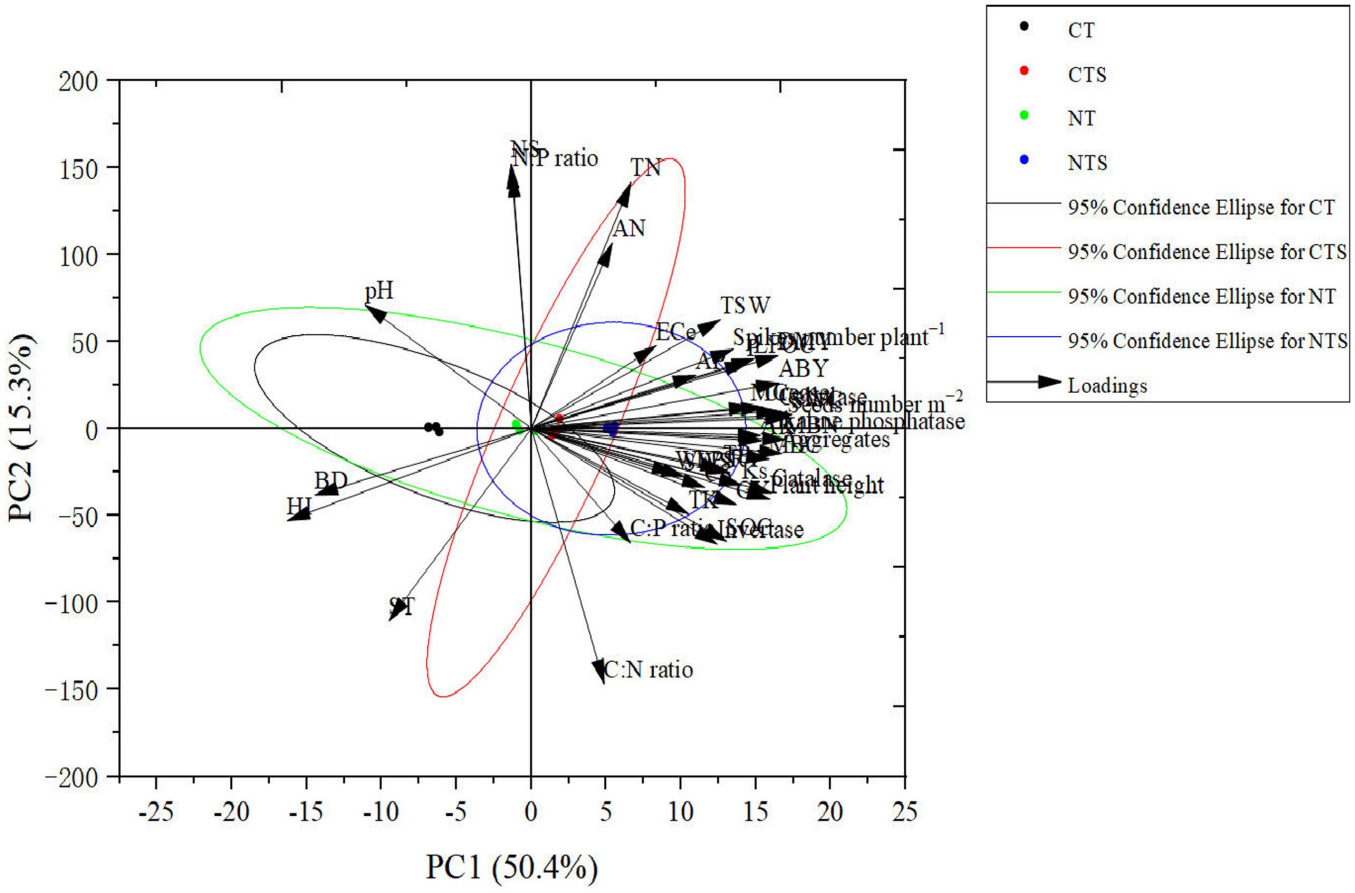
Principal component analysis of spring wheat agronomic attributes and soil properties (physical, chemical, and biological).

**Figure 5:**
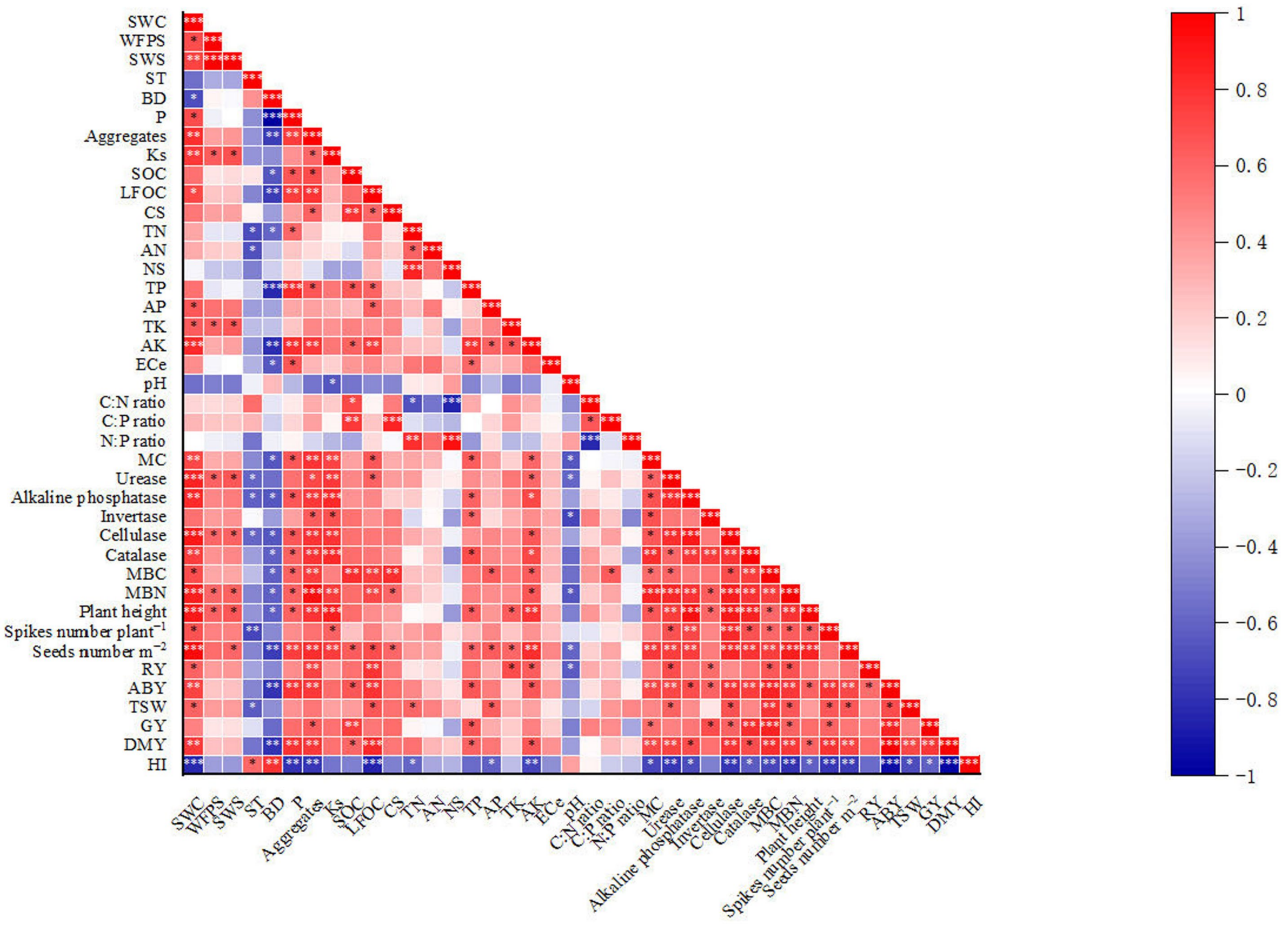
Heat map correlation study of wheat agronomic traits and soil physical, chemical, and biological properties. Indicates significance at: *p < 0.05, **p < 0.010, and ***p < 0.0010. Note: the abbreviated words stand for SWC = soil gravimetric water content; WFPS = water-filled pore space; SWS = soil water storage; ST = soil temperature; BD = soil bulk density; P = soil porosity; Ks = soil saturated hydraulic conductivity; SOC = soil organic carbon; LFOC = light fraction organic carbon; SC = carbon storage; TN = total nitrogen; AN = available nitrogen; NS = nitrogen storage; TP = total phosphorous; AP = available phosphorous; TK = total potassium; AK = available potassium; ECe = soil electrical conductivity; pH = soil pH; MC = soil microbial counts; MBC = microbial biomass carbon; MBN = microbial biomass nitrogen; RY = wheat root yield; ABY = wheat aboveground biomass yield; TSW = thousand seed weight; GY = wheat grain yield; DMY = dry matter yield; HI = harvest index.

Furthermore, the principal component analysis (PCA) in accordance with the Jolliffe cutoff value permits isolating five principal components. In PCA analysis, the observation point made by contact between PC1 and PC2 illustrates the general variance defined by the five chief components. The PCA analysis of 39 variables (namely SWC, WFPS, SWS, ST, BD, P, soil aggregates, Ks, SOC, LFOC, CS, MBC, TN, AN, NS, MBN, TP, AP, TK, AK, ECe, pH, C:N ratio, C:P ratio, N:P ratio, MC, urease, alkaline phosphatase, invertase, cellulase, catalase, plant height, root yield, number of spikes per plant, number of seeds per meter square, grain yield, aboveground biomass yield, dry matter yield, harvest index), PC1 and PC2 were extracted with eigenvalues >1 and explained 65.7% of the total variance. Nevertheless, PC3, PC4, and PC5 do not allow the addition of additional information; that is why they are not involved. The highest PC1 loadings encompassed 50.4% of the total variance, and in PC2, the maximum loadings of 15.3% of the total variance were detected (Figure 4). According to our expectations, the correlation between wheat seed productivity and yield-attributing traits was significantly positive. Moreover, a significant positive correlation was noted between soil hydraulic properties, soil nutrients, and enzymatic activities and the seed yield of spring wheat (Figure 5). This concurs with the results reported by Yuan et al. (2023a).

### 3.3 Greenhouse gas fluxes and drivers of GHG emissions

**Figure 6:**
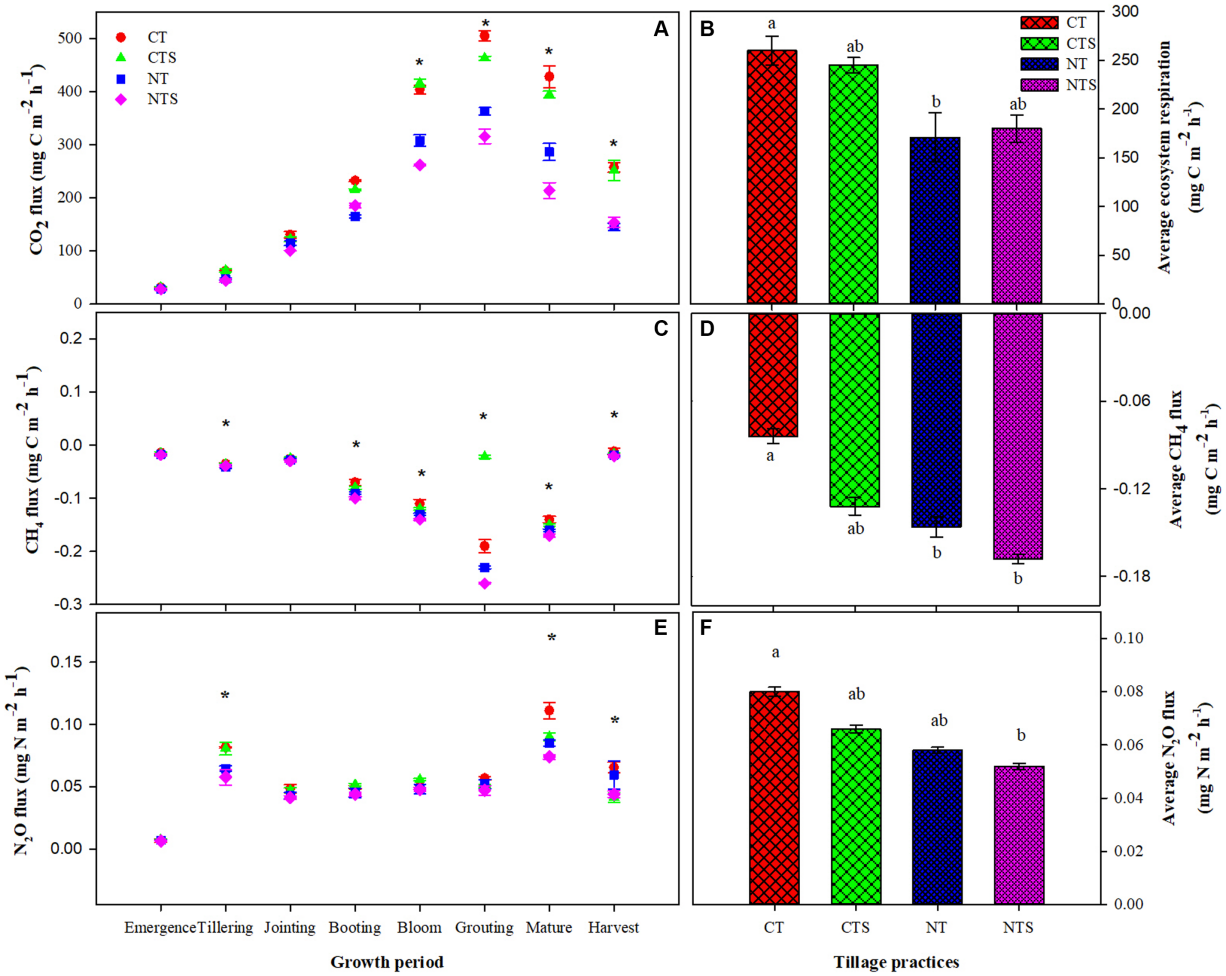
Seasonal and average greenhouse gas emissions under tillage systems in spring-wheat agroecosystem in 2021. Error bars represent the corresponding standard error of mean values; *n* = 3. Different lower-case letters and ‘*’ indicate significant differences amongst different treatments at *p* < 0.05 (Duncan’s test performed for mean separation). Note: **(A,B)** is the seasonal and average ecosystem respiration as affected by the tillage treatments; **(C,D)** is the seasonal and average CH_4_ flux under conservation tillage; **(E,F)** is the seasonal and average N_2_O flux under tillage practices.

Significant variation in GHG (CO_2_, CH_4_, and N_2_O) was observed under different tillage regimes (Figure 6). The efflux rates of CO_2_ (representing ecosystem respiration) under conservation tillage depicted apparent seasonal variations (Figure 6A). The maximum emissions arising during the summer months peak in the CT system in July, at the grouting spring wheat crop growth stage. The CT and CTS treatments showed the highest ecosystem respiration rates, including all investigated crop growth stages, while the NTS and NT treatments had the lowest CO_2_ emission rates, but a significant difference between the different treatments was observed in bloom, grouting, maturity, and harvest stages. The highest CO_2_ efflux rates during the summer months correspond with the maximum ST and dry matter accumulation, hence augmented soil respiration (Ussiri et al., 2009). Wu et al. (2011) clarified that an increase in soil temperature from 5°C to 15°C led to a noteworthy increase in the emission of carbon dioxide because of high soil organic matter mineralization and improved soil microbial activity. Lowering of soil CO_2_ efflux rates during the growing seasons under conservation tillage was previously reported by many researchers (Alhassan et al., 2021; Li et al., 2023). Al-Kaisi and Yin (2005) stated a huge 80% reduction in CO_2_ efflux rates under the NT compared to the CT system. Average CO_2_ efflux rates were higher in tilled soils compared with non-tilled soils. The average CO_2_ efflux rates were 180 ± 14.2, 171 ± 25.8, 245 ± 8.8 and 260 ± 15.3 mg Cm^−2^ h^−1^ in NTS, NT, CTS and CT respectively (Figure 4B). A significant positive correlation of CO_2_ emission among soil moisture and soil temperature and a negative correlation of CO_2_ emission with nitrogen and carbon contents and hydraulic conductivity were observed during the current study (Table 6). The CO_2_ efflux rates are habitually controlled by plenty of factors, including carbon dioxide concentration gradient in the environment and the wind speed, soil water, soil temperature, soil medium, and soil physicochemical properties (Raich and Schlesinger, 1992). The tillage effect on these parameters would affect carbon dioxide emissions as well. The CT causes soil disturbance, which increases rates of decomposition because of improved soil microbe activities (Al-Kaisi and Yin, 2005), leading to maximum emissions of CO_2_. Quite the reverse, under NTS and NT treatments, decomposition is slower because of no soil disturbance (Curtin et al., 2000). Conservation tillage practices (CTS, NT, and NTS) might correspondingly improve soil properties, which in turn can decrease soil CO_2_ emissions. Maximum SWC under CTS treatment, combined with maximum ST, produced higher cumulative soil CO_2_ emissions compared with NT and NTS. The SWC and ST habitually exert a collaborative effect on CO_2_ emissions (Bond-Lamberty et al., 2016).

**Table 6:** Heat map correlation study of greenhouse gases and soil indicators under tillage systems.

Variables		Greenhouse gases		
	CO_2_	CH_4_	N_2_O	GWP
SWC	0.95*	0.98*	0.97*	0.96*
WFPS	0.96*	0.97*	0.95*	0.99*
ST	0.97*	0.96*	0.95*	0.98*
BD	0.88	0.84	0.83	0.85
Ks	−0.98*	−0.95*	−0.97*	−0.96*
SOC	−0.86	−0.84	−0.83	−0.85
LFOC	−0.85	−0.82	−0.79	−0.83
TN	−0.75	−0.73	−0.77*	−0.81*
AN	−0.72	−0.70	−0.68	−0.66
MBC	−0.82	−0.76	−0.67	−0.95*
MBN	−0.84	−0.72	−0.78	−0.71

Indicates significance at: *p < 0.05, **p < 0.010, and ***p < 0.0010. The abbreviated words stand for SWC, soil moisture; WFPS, water-filled pore space; ST, soil temperature; BD, soil bulk density; Ks, soil saturated hydraulic conductivity; SOC, soil organic carbon; LFOC, light fraction organic carbon; TN, total nitrogen; AN, available nitrogen; MBC, microbial biomass carbon; MBN, microbial biomass nitrogen; CH_4_, methane; CO_2_, carbon dioxide; N_2_O, nitrous oxide; GWP, global warming potential.

All the tested tillage systems acted as CH_4_ sinks during the study period (Figure 6C). Peak absorption arose during the summer months at booting and bloom crop growth stages under the NT system. Seasonal changes were recorded in the sink capacities of different tillage systems. The CT had minimum CH_4_ absorption rates during the investigated crop growth stages. A significant difference between different treatments was observed except for emergence and jointing of spring wheat crop growth stages. The maximum CH_4_ emissions during the summer months were because of the high SWC status and ST, which may perhaps have boosted the activity of CH_4_-oxidizing bacteria. The analysis of variance showed that all the investigated tillage techniques, namely CT, CTS, NT, and NTS, had a significant influence on average methane emission. In general, our results showed the average absorption rate of CH_4_ followed the trend of CT < CTS < NT < NTS (Figure 4D). Another study done by Yeboah et al. (2016) had comparable consequences. Shen et al. (2018) showed that semi-arid agroecosystems habitually act as sinks of CH_4_ as a result of soil aerobic conditions (Schaufler et al., 2010). Similar to the emission of CO_2_, the absorption has a significant positive correlation with ST and SWC and a negative correlation with Ks and carbon and nitrogen elements (Table 6). The results of the current study are consistent with the results recorded by Yeboah et al. (2016) and Wang et al. (2022), who stated that improved SWC status can lead to high CH_4_ uptake owing to the anaerobiosis occurrence and methanogenesis increment. Singh et al. (2010) observed maximum CH_4_ emission rates under 13% soil water content in sandy loamy soils, and a parallel situation was detected in this study. Our study depicted that the methane flux was constantly negative, signifying CH_4_ uptake by all the tillage systems. The NTS had the highest CH_4_ uptake in this study. Under NTS treatment, minor ST might have played a substantial role in high CH_4_ uptake. During high ST, the dominant methanogen (Methanosarcinaceae) employs H_2_/CO_2_ and acetate as precursors of CH_4_ producing and produces far maximum CH_4_ over the methanogen at lesser temperatures (Methanosaetaceae), which uses solitary acetate as a precursor of CH_4_ producing (Ding et al., 2003). Our results are consistent with other appraisals in the Loess Plateau of China, where NT with straw-return was found to be a net sink for atmospheric CH_4_ (Alhassan et al., 2021). The greater methane uptake under the NTS system might be because of good soil aeration and less degradation of the soil, which improved the activity of methanotrophs. McLain and Martens (2006) recorded that the activity of methanotrophs was heightened under tolerable gas diffusion and from the microbial activity sites. The NTS system inclines to increase soil organic carbon and decrease the bulk density of soil, which might lead to lesser CH_4_ emissions risk. Yeboah et al. (2016) stated that the degradation of soil can diminish the soil’s ability to oxidize or consume atmospheric methane by as much as 30–90%. However, we proved that the most noteworthy CH_4_ uptake control was SWC at the surface soil depth, with ST and Ks brought about by enhanced soil condition or quality and improved SOC. Soil carbon and nitrogen contents are also negatively correlated with CH_4_ emission. This obscures the fact that improved soil carbon and nitrogen levels condensed the emission of CH_4_.

Our study data showed that peak N_2_O emission occurred in August at the final crop maturity growth stage, while the lowest N_2_O flux was observed in the spring months. This trend is consistent with the findings stated by Cai et al. (2013) at the identical research site. During most tested crop growth stages, the CT and CTS emit more N_2_O compared with the NT and NTS treatments, while at the harvest crop growth stage, NT served as a slight emitter and dominated the CTS system. A significant difference between different treatments was observed only at tillering, maturity, and harvest crop growth stages (Figure 6E). The N_2_O flux pattern depicted that when SWC and ST appeared higher during the summer season, the N_2_O emissions were higher. These consequences are similar to earlier studies (Bhattacharyya et al., 2023; Bozal-Leorri et al., 2023; Li et al., 2023). The ST is a significant factor influencing seasonal variations of N_2_O flux. Taghizadeh-Toosi et al. (2022) observed that the variation in N_2_O flux rate is almost harmonized with the surface temperature of the soil. Zhang et al. (2022) noted that ST increasement can diminish the nitrification contribution to N_2_O and increase the N_2_O amount produced during the denitrification process. The highest N_2_O emissions noted during the rainiest months under all tested tillage systems might be credited to the fact that the spell’s rainfall levels might be higher than normal on that date, making all tillage systems very wet, so that denitrification situations were not dissimilar among them.

Significantly higher N_2_O fluxes were observed under CT treatment compared with NTS, NT, and CTS treatments. The N_2_O flux rates followed the trend of NTS < NT < CTS < CT (Figure 4F). On average, all investigated tillage systems served as slight N_2_O emitters. These results are in accordance with those described by Pokharel and Chang (2021). Quite the reverse, Alhassan et al. (2021) observed maximum N_2_O fluxes under the NT system over the CT technique. In this study, different conservation tillage practices, for instance, CTS, NT, and NTS, decreased the emission of N_2_O by increasing soil structure, as revealed by bulk density of soil, pore space, soil aggregates, and soil hydraulic conductivity. Higher N_2_O emissions under the CT system have been credited to the condensed diffusivity of gas and air-filled pore space (Rabot et al., 2015). Additionally, N_2_O flux rates recorded in this experimental research were in the range of N_2_O flux rates noted by Yeboah et al. (2016).

A significant positive correlation was noted between ST and N_2_O emissions, which is attributed to the microbial respiration augmentation after heating origins oxygen dearth in the soil profile, which generates anaerobic conditions for denitrifying microbes’ activity (Castaldi, 2000; Mehnaz et al., 2018), and all together, warming also rises soil denitrification activity (Braker et al., 2010), which led to a rise in N_2_O emissions. Additionally, our results also showed positive correlations among SWC and N_2_O emissions. This is due to the improvement in SWC that limits the soil oxygen concentration, chiefly the anaerobic soil environment formation, pointedly falling nitrification and increasing denitrification, which in turn led to dominant denitrification to produce N_2_O emissions (Pokharel and Chang, 2021). Our results are identical with other scientific reports by Taghizadeh-Toosi et al. (2022), demonstrating the influence of ST and SWC on the emission of soil N_2_O.

The correlation study in Table 6 showed a significant negative relationship between N_2_O emission and carbon and nitrogen contents, supporting the (He et al., 2023) that enhancing carbon stocks and nitrogen soil status can decrease the emission of soil N_2_O. Consequently, these findings are consistent with earlier results reported by Zhao et al. (2020). High soil bulk density related to the degradation of soil by tillage application can have consequences for soil aeration reduction. Soil degradation reduction, whereas surface soil preservation covered by crop straws, might lead to minor denitrification as well as N_2_O emissions risk. Quite the reverse, Li et al. (2005) reported a positive correlation between soil N_2_O emissions and soil carbon contents. This is due to the increase in soil carbon contents delivering sufficient sources of nitrogen and a suggestive rise in the heterotrophic microorganism’s respiration. All at once, carbon availability in the soil offers electron donors for denitrifying microbes, which endorses the denitrification incidence, in that way increasing emissions of N_2_O. Soil nitrogen contents were positively correlated with N_2_O emission. Su et al. (2021) stated that improved N_2_O production might be a consequence of augmented nitrogen contents and bacterial activities; meanwhile, the activities of microbes are controlled by soil carbon contents. This obscures that soil carbon contents improve the N_2_O-producing microbe’s activities. This also verifies the results of Guenet et al. (2021), who showed that high carbon contents mostly raise the emission of N_2_O. Changes in CO_2_, N_2_O, and CH_4_ emission seemed to be described by variations in soil temperature, moisture content, and saturated hydraulic conductivity in the surface soil layer of 0–10 cm soil depth, with 95% of the data variance described by these environmental variables.

### 3.4 Global warming potential under conservation tillage system

**Figure 7:**
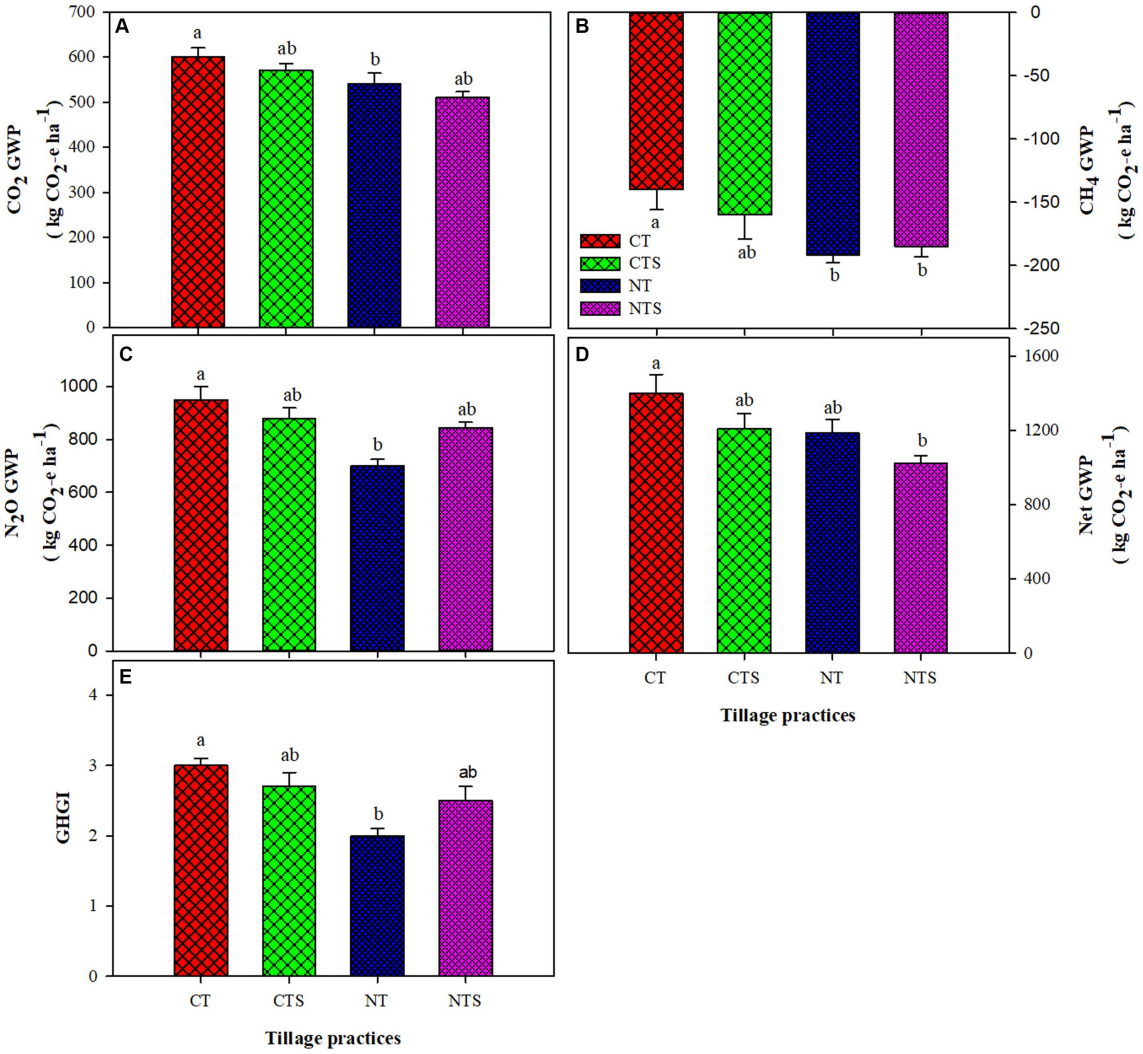
Global warming potential (GWP) of CO_2_, CH_4_ and N_2_O and greenhouse gas intensity (GHGI) under tillage practices in spring-wheat agroecosystem in 2021. Error bars represent the corresponding standard error of mean values; *n* = 3. Different lower-case letters and ‘*’ indicate significant differences amongst different treatments at *p* < 0.05 (Duncan’s test performed for mean separation). Note: **(A)** is the GWP of carbon dioxide as affected by the tillage treatments; **(B)** is the GWP of CH_4_ as influenced by the tillage system; **(C)** is the GWP of N_2_O as affected by the different tillage techniques; **(D)** is the net-GWP under tillage measures and **(E)** is the GHGI under tillage practices.

**Figure 8:**
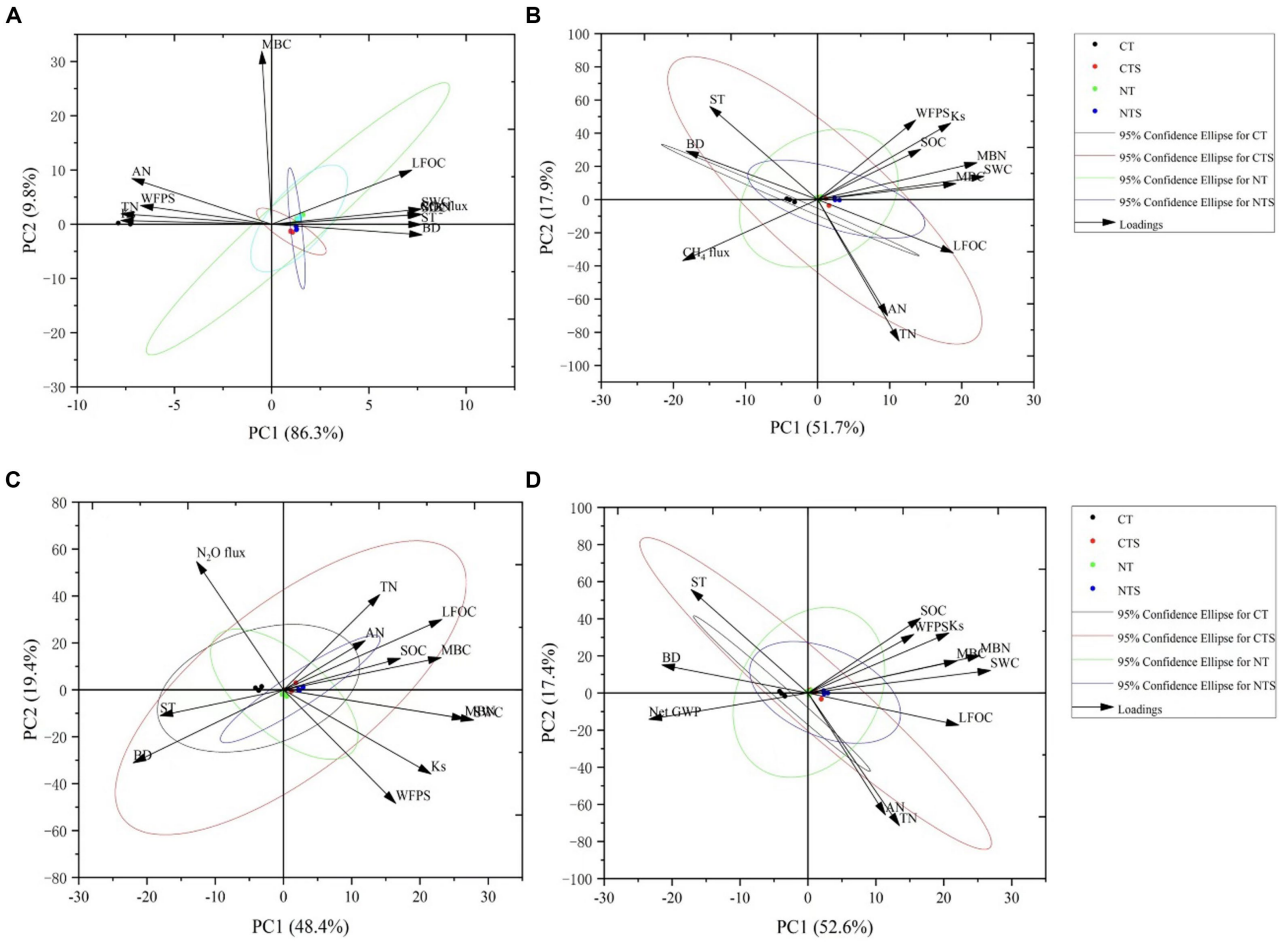
Principal component analysis for evaluating the influence of environmental variables on greenhouse gas emissions under conservation tillage practices. **(A)** The PCA analysis of environmental variables with carbon dioxide emission; **(B)** the PCA analysis of environmental variables with methane emission; **(C)** the PCA analysis of environmental variables with nitrous oxide emission; and **(D)** the PCA analysis of environmental variables with net global warming under different tillage systems.

The GWP of CO_2_, CH_4_ and N_2_O and net global warming potential (GWP) under all investigated tillage practices are shown in (Figure 7). The highest cumulative CO_2_ flux was recorded under CT, whereas conservation tillage measures NTS, NT, and CTS pointedly reduced the cumulative CO_2_ flux, as the least cumulative CO_2_ flux was noted under NT, which was followed by NTS. The global warming potential due to the emissions of CH_4_ was significantly lower under NTS, which was followed by NT over CT during the study period. The global warming potential due to the emissions of N_2_O was significantly less under NT, which was followed by NTS over CT. The net global warming potential followed the trend of CT > CTS > NT > NTS (Figure 7D). The NTS, NT, and CTS decreased net-GWP by 23.44, 19.57, and 16.54%, respectively, over the CT system. The effect of different tillage practices on the atmospheric radiative forcing and henceforth changing climate can be evaluated by global warming potential determination from the biosphere–atmosphere exchange of numerous greenhouse gases. The noted global warming potential for the current study ranged between 1000 and 1400 kg CO_2_-e ha^−1^ which is in the range of those stated by Ma et al. (2013) but superior to those stated by Yeboah et al. (2016). The net-GWP was positively correlated with ST and SWC and negatively correlated with carbon and nitrogen elements and Ks (Table 6). In PCA analysis, we found that soil moisture, soil temperature, bulk density, hydraulic conductivity, carbon, and nitrogen elements explained 96.2, 69.6, 67.8, and 77% of the variations in soil CO_2_, CH_4_, and N_2_O fluxes and GWP, respectively (Figures 8A–D). Compared with CH_4_ and N_2_O fluxes and GWP, soil CO_2_ had a strong relationship with environmental variables, as shown in Figure 8A. Conservation agriculture has the potential to contribute to environmental conservation, though its influence differs depending on management strategies. The higher greenhouse gas intensity (GHGI) was associated with the CT system, while the minimum GHGI value was observed with the NT system, which was followed by the NTS system. The GHGI followed the trend of CT > CTS > NTS > NT. The conservation tillage systems NT, NTS, and CTS decreased GHGI by 29.96, 23.20, and 18.72%, respectively, compared with CT practice (Figure 7E). The current noted global warming intensity values were higher compared with other studies (Qin et al., 2010; Ma et al., 2013), which is attributable to the overall lower grain yield in this experimental research. Global warming intensity is a crop yield function, and it is directly affected by low grain yield. A significant reduction was observed under NTS and NT practices over CTS and CT systems in case of global warming potential and global warming intensity values. These findings are in line with Alhassan et al. (2021) and Li et al. (2023), who stated that different conservation tillage systems decreased the global warming potential and global warming intensity compared with the CT technique.

## 4 Conclusion

Conservation tillage is a significant climate-smart approach to increasing soil and water conservation, soil carbon sequestration, crop production and reducing greenhouse gas emissions. It is argued to decrease global warming potential in relation to changing climate mitigation. Our study suggests that conservation tillage practices CTS, NT, and NTS have the potential to increase wheat agronomic traits (plant height, number of spikes per plant, seeds number per meter square, root yield, aboveground biomass yield, thousand-grain weight, grain yield and dry matter yield), soil physical properties (gravimetric soil water content, water-filled pore space, water storage, pore space, aggregates, and hydraulic conductivity), chemical properties (soil organic carbon, light fraction organic carbon, carbon storage, total nitrogen, available nitrogen, nitrogen storage, total phosphorous, available phosphorous, total potassium, available potassium), biological properties (soil microbial count, urease, alkaline phosphatase, invertase, cellulase, catalase, microbial biomasses carbon and nitrogen) and decrease carbon, methane and nitrous oxide emissions in semi-arid sandy loam cropland of Dingxi China in comparison with plowing conventional tillage system. Moreover, NTS and NT significantly reduced the global warming potential of carbon dioxide, methane, and nitrous oxide and their yield-scale global warming potential. The correlation analyses show that crop yield is significantly positively correlated with yield-attributing traits, soil physicochemical properties, and biological properties. The global warming potential due to CO_2_, CH_4_, and N_2_O greenhouse gases had a positive correlation with soil and environmental variables, and the dominant controlled factors were soil moisture and soil temperature. Accordingly, straw-retention with no tilled soil and stubble incorporation with conventionally tilled soil should be recommended and promoted among the smallholder farmer systems in semi-arid zones to raise soil and water conservation, soil and environmental quality, and sustainability. More comprehensive long-term research and different tillage operations are needed regarding carbon sequestration and stocks and genes involved in nitrogen cycling and global warming to produce more powerful data for climate-smart agriculture.

## Data availability statement

The raw data supporting the conclusions of this article will be made available by the authors, without undue reservation.

## Ethics statement

The studies involving humans were approved by College of Forestry, Gansu Agricultural University, Lanzhou 730,070, China; khanmahran420@gmail.com (MS). The studies were conducted in accordance with the local legislation and institutional requirements. The ethics committee/institutional review board waived the requirement of written informed consent for participation from the participants or the participants’ legal guardians/next of kin because College of Forestry, Gansu Agricultural University, Lanzhou 730,070, China; khanmahran420@gmail.com (MS).

## Author contributions

MS: Conceptualization, Data curation, Formal analysis, Methodology, Resources, Software, Validation, Writing – original draft, Writing – review & editing. NR: Writing – review & editing, Writing – original draft. MT: Writing – review & editing, Writing – original draft. ARA: Writing – review & editing, Conceptualization, Methodology, Writing – original draft. MMA: Writing – review & editing, Conceptualization, Data curation, Methodology, Writing – original draft. ALA: Writing – review & editing, Conceptualization, Formal analysis, Methodology, Validation, Writing – original draft. KZG: Funding acquisition, Resources, Writing – review & editing, Data curation, Formal analysis, Investigation, Visualization, Writing – original draft. MAA: Writing – review & editing, Data curation, Investigation, Software. MA: Writing – review & editing, Conceptualization, Formal analysis, Methodology. NM: Writing – review & editing, Conceptualization, Data curation, Writing – original draft. JY: Writing – review & editing. AS: Writing – review & editing. MS: Writing – review & editing, Writing – original draft. MHE: Funding acquisition, Writing – original draft, Data curation, Formal analysis, Writing – review & editing. GC: Writing – review & editing. GL: Formal analysis, Funding acquisition, Project administration, Resources, Supervision, Visualization, Data curation, Methodology, Writing – original draft, Writing – review & editing.
